# Characterization of the Mucosally-Adherent Duodenal Microbiome in Children with and without Crohn’s Disease

**DOI:** 10.3390/ph15070850

**Published:** 2022-07-11

**Authors:** Kenneth Schmidt, Janelle Noel-MacDonnell, Carrie Vyhlidal, Daniel P. Heruth, Vivekanand Singh, Atif A. Ahmed, Taina Hudson, Veronica Williams, Valentina Shakhnovich

**Affiliations:** 1 School of Medicine, University of Missouri Kansas City, Kansas City, MO 64108, USA; k.c.schmidt7@gmail.com (K.S.); jrnoelmacdonnell@cmh.edu (J.N.-M.); dpheruth@cmh.edu (D.P.H.); tjhudson@cmh.edu (T.H.); vwilliams@cmh.edu (V.W.); 2 Children’s Mercy Kansas City, Kansas City, MO 64108, USA; 3 KCAS Bioanalytical & Biomarker Services, Shawnee, KS 66061, USA; carrie.vyhlidal@kcasbio.com; 4 Department of Pathology, University of Texas Southwestern Medical Center, Dallas, TX 75390, USA; vivekanand.singh@utsouthwestern.edu; 5 Seattle Children’s Hospitals, Seattle, WA 98105, USA; atif.ahmed@seattlechildrens.org

**Keywords:** inflammatory bowel disease, Crohn’s disease, duodenum, microbiome, pediatric, small intestine

## Abstract

Manipulation of the microbiome is a rational treatment strategy for inflammatory bowel disease (IBD). Compared to the colon and terminal ileum (TI), understanding of the microbial composition in the duodenum is sparse. This gap in knowledge is especially significant for children with Crohn’s disease (CD) because the prevalence of duodenal CD is higher in children than in adults. Our aim was to characterize the bacterial composition of the mucosally-adherent duodenal microbiome in children with and without CD as a first step toward development of targeted IBD treatment strategies at this disease location. Fresh-frozen mucosal biopsies were obtained from the duodenum and TI of children with treatment-naïve CD and age- and sex-matched controls. Extracted DNA was analyzed for sequence variation in the 16S ribosomal RNA bacterial gene region V4 (Novogene; Beijing, China). Bacterial relative abundance, alpha and beta composition, and diversity, were compared across duodenal and TI samples from the controls and CD groups with and without chronic active inflammation (118 samples from 73 children total; approx. 50% CD), using UniFrac dissimilarity coefficients (α < 0.05), Linear Discriminant Analysis Effect Size (LEfSe) analysis (LDA score ≥ 2), and Unweighted Pair Group Method with Arithmetic Mean (UPGMA) analysis. The relationships between bacterial abundance, sex, age, concomitant medication use, and villous length were assessed. The microbial composition in the duodenum was significantly different from the TI in the control population(R-value = 0.558, *p* = 0.001) and in children with active CD (R-value = 0.301, *p* = 0.001). Significant differences in bacterial abundance were noted between the control and CD duodena (LDA > 4). The duodenum of children without CD was characterized by increased abundance in Pseudomonodales, whereas the actively inflamed duodenum in CD was characterized by increased abundance of Bacteroidales, specifically the family Prevotellaceae. This trend is opposite of previously published observations of microbial composition in the TI, where active inflammation was associated with a relative decrease in the abundance of Bacteroidetes and an increase in Proteobacteria, including Pseudomonadales. No statistically significant correlations were noted between abundance and age, sex, concomitant medication use or villous length, except for Bacteroidetes, which significantly decreased in abundance in the TI with age (*p* = 0.048). The pediatric duodenal microbiome is distinct from the TI and characterized by an increased abundance of Pseudomonodales and Spirochetes in healthy children, and an increased abundance of Bacteroidales in active CD patients.

## 1. Introduction

Inflammatory Bowel Disease (IBD), with its major subtypes Crohn’s disease (CD) and Ulcerative Colitis (UC), is a chronic disease of the gastrointestinal (GI) tract, marked by relapsing and remitting episodes of inflammation of the intestinal mucosa [1,2]. Although its exact etiology remains unknown, the consensus of the pathogenic model consists of a multifactorial, dynamic dysregulation of the host immune system, genetic predisposition, environmental triggers and dysbiosis of the intestinal microbiome [3]. Progress in understanding the role of each of these domains has been variable, and the microbiome remains comparatively poorly understood, even though its manipulation has been identified as a rational treatment strategy for IBD [4].

What is currently known is that the neonatal GI tract is initially colonized with flora from the mother in-utero and/or during birth [5]. The microbiome subsequently develops over the ensuing years, primarily in response to environmental exposures [6]. This chronological development, in combination with altering effects of other exogenic influences such as diet, medication use, and acute and chronic illness, creates a dynamically changing microbial composition that has proven somewhat difficult to characterize comprehensively across the pediatric age range, as well as within specific patient populations.

To date, most studies in IBD have focused on the colon and the distal small intestine (i.e., terminal ileum), where bacteria are most abundant. However, the proximal small intestine (i.e., duodenum) is not sterile, and unique differences in bacterial composition can be anticipated because different locations within the small intestine have been shown to have different microbial compositions [7]. Understanding of the bacterial composition in the pediatric duodenum is particularly important because duodenal pathology is more common in pediatric onset CD, compared with adult-onset disease [8,9,10,11], raising the possibility that dysbiosis may play a role in duodenal disease manifestation and/or treatment. Yet, to our knowledge, no studies exist regarding the duodenal microbial composition in pediatric IBD during disease remission vs. flare. Given this knowledge gap, we aimed to characterize the bacterial composition of the duodenal microbiome in children with and without CD, with and without active duodenal disease involvement. To highlight findings specific to the duodenum, we also sequenced the terminal ilea from these children for comparison.

## 2. Results

### 2.1. Participants and Samples

In total, 146 small intestinal mucosal biopsy samples from 75 children were sent to Novogene for 16S sequencing of the bacterial V4 region. Of these, 118 samples from 73 children passed initial quality control (QC) and were sufficiently free of contamination from human DNA. The QC pass rate for samples from the duodenum was lower (64%) than for the TI (93%). Ultimately, 48 duodenal samples (27 from controls and 21 from the CD group (8 active duodenal disease, 13 inactive duodenal disease) and 68 TI samples (33 from controls and 35 from the CD group (29 active TI disease, 6 inactive TI disease) were available for analysis. Duodenal and TI samples from one child were specifically excluded from statistical analysis due to discovery of concomitant acute enteric infection at the time of study biopsy collection.

### 2.2. Characterization of the Duodenal Microbiome

As summarized in Table 1, the duodenal patient population (Duo) was comprised primarily of adolescents. There were more samples from males than females across all three Duo study groups: Control (Duo0), Active Duodenal CD (Duo1), and Inactive Duodenal CD in the presence of active CD elsewhere (Duo2). However, differences in gender composition did not reach statistical significance and no statistically significant differences in age were observed across the study groups.

As expected, overall concomitant medication use at the time of biopsy collection was consistently higher in the CD group than in the controls (presumably for symptom control prior to diagnosis), but this difference did not reach statistical significance (Table 1). No children were receiving treatment with immunomodulating drugs at the time of biopsy, and only one child with CD was receiving treatment with aminosalicylates. Closer inspection of the use of concomitant medications that could potentially alter bacterial composition in the intestine (i.e., antibiotics, probiotics, and acid suppression medications) revealed no statistically significant difference between the CD group and controls. However, a trend in higher probiotic and antibiotic use was identified in the CD group compared to controls (Table 1). None of the children enrolled were following any specific diet recommendations for CD.

As summarized in Table 2, the overall microbial composition in Duo0 was statistically significantly different from the composition in the TI for the controls (TI0) (R-value = 0.558, *p* = 0.001) and CD-Active (TI1) (R-value = 0.301, *p* = 0.001), but not CD-Inactive (TI2) (R-value = 0.123, *p* = 0.1). Similar trends were also observed between the Duo1 and Duo2 populations compared with TI0 and TI1. Although no significant differences in the overall bacterial composition within the duodenal microbiome were noted in controls vs. children with CD (Table 2), within the three duodenal study groups (Duo0, Duo1 and Duo2), certain bacterial operational taxonomic units (OTUs) were noted to be significantly dissimilar in terms of levels of relative abundance.

**Figure 1:**
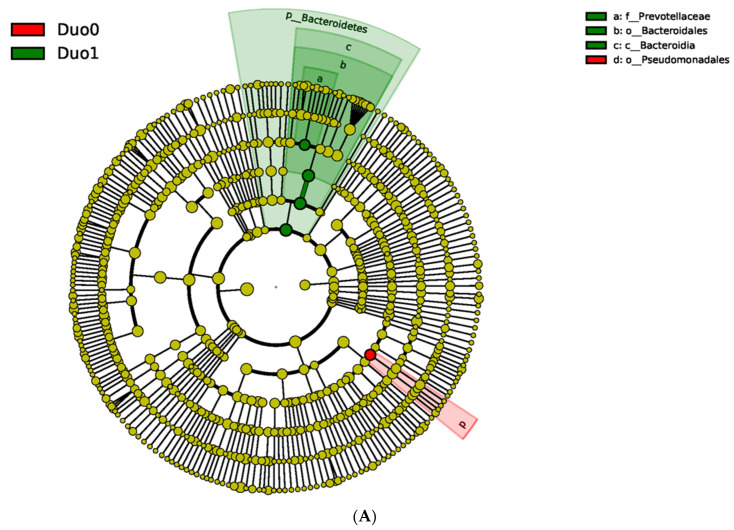

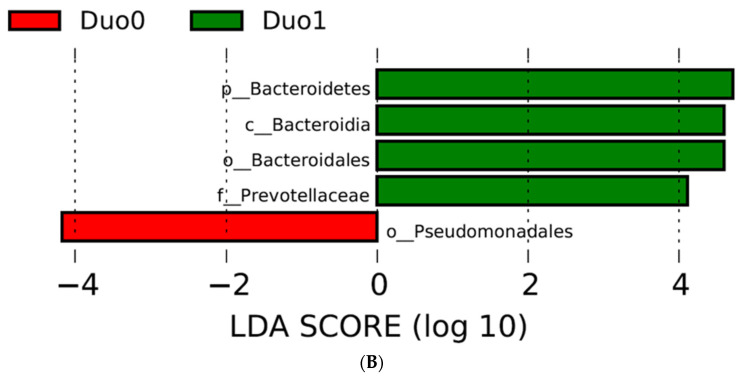
Linear Discriminant Analysis effect size (LEfSe) comparing relative bacterial composition in the duodenum between controls (Duo0, red) and children with active Crohn’s disease in the duodenum (Duo1, green). (**A**) The cladogram compares differences between bacterial entities in the duodena of controls and children with active Crohn’s Disease (CD). Innermost nodes represent the phylum level of classification and move outward to class, order, family and genus. Yellow nodes denote bacterial entities that were not significantly different between the two duodenal study groups. (**B**) Bar graph with log 10 transformed Linear Discriminant Analysis (LDA) scores that numerically compares the most abundant bacterial taxa between controls and children with active duodenal CD.

Specifically, the order Pseudomonadales was noted to be significantly more abundant in controls compared with children with active CD, who demonstrated a significant increase in abundance of the phylum Bacteriodetes, and more specifically the family Prevotellaceae, as defined by a Linear Discriminant Analysis (LDA) score > 4 (Figure 1).

**Figure 2:**
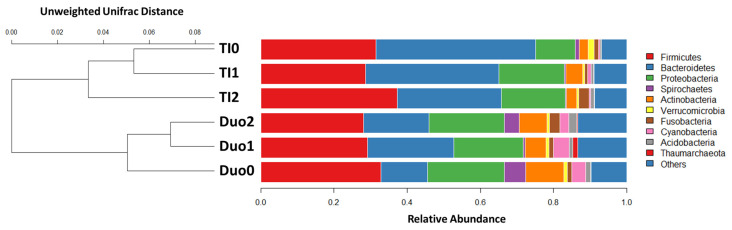
Unweighted Pair-group Method with Arithmetic Mean (UPGMA) analysis to assess and compare alpha and beta diversity across tissues and study groups. The bar chart depicts the relative abundance of bacteria at the phyla level for both the duodenum (Duo) and terminal ileum (TI). The dendrogram on the left allows comparison across tissues and study groups, highlighting the largest degree of difference between Duo and TI, Duo0 & TI0-Controls, Duo1 & TI1-Active CD at that biopsy site, and Duo2 & TI2-CD without active inflammation at that biopsy site, but present elsewhere.

These observations were supported in an Unweighted Pair Group Method with Arithmetic mean (UPGMA) analysis assessing alpha and beta diversity in the duodenum (Figure 2), where we observed an 11% increase in Bacteriodetes abundance in the duodena of children with active CD compared with the controls. In contrast, the control duodenal microbiome appeared to be characterized by an increased abundance of Spirochetes (*p*-value = 0.28); however, the LDA criteria for statistical significance was not reached for either Spirochetes or Bacteriodetes in this analysis.

To further explore whether concomitant medication use could explain some of the differences in OTU abundance observed in the duodena of children with CD vs. controls (Figure 1), we compared OTU abundance between children receiving and not receiving concomitant medications within each duodenal study subgroup (Duo0, Duo1 and Duo2). No statistically significant differences were observed (Table 3).

### 2.3. Characterization of the TI Microbiome

The patient characteristics of the TI population (Table 4) were similar to the Duo population, with slightly more males than females (not statistically significant) and no statistically significant differences in age across the three study groups: Control (TI0), Active TI CD (TI1), and Inactive TI CD with presence of active CD elsewhere (TI2). More patients were included in the TI population than the Duo population, as more tissue samples passed quality control for library preparation.

The reported medication use at time of endoscopy was significantly higher in CD patients (Table 4). Individual use of probiotics, antibiotics, acid suppressants, or aminosalicylates (CD only) was not significantly different across the three TI groups. No children were receiving treatment with systemic immunomodulators at the time of biopsy, but one child with CD-active (TI1) was receiving concomitant therapy with budesonide, a locally acting steroid (Table 4).

**Figure 3:**
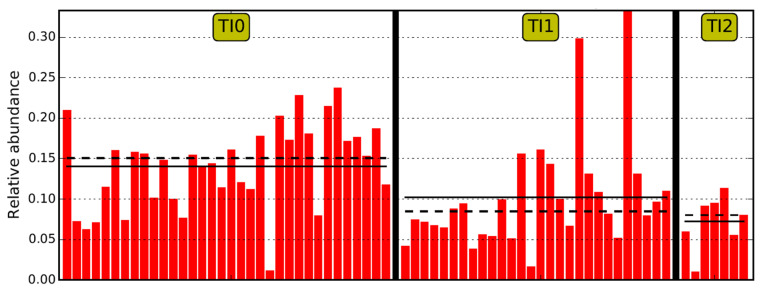
Comparison of the mean (dashed horizontal line) and median (solid horizontal line) abundance of *Lachnospiraceae* in TI in controls (TI0), active CD (TI1) and inactive CD (TI2).

Comparing the TI microbiome Operational Taxonomic Unit (OTU) relative abundance between the control and CD populations, the class of *Clostridia* and, more specifically, the family of *Lachnospiraceae* were significantly increased in the TI of the controls (Figure 3). Using LEfSe analysis, no OTUs met the threshold for significance within the CD active (TI1) and inactive (TI2) populations (LDA score > 4).

### 2.4. Comparison of Duodenal and TI Microbiomes

As previously mentioned, significant differences in the overall bacterial composition were noted between the duodenum and TI in both the controls and in children with active CD; however, no significant differences were noted when comparing the duodenum to the TI of children with Crohn’s but no active inflammation in the TI (Table 2).

**Figure 4:**
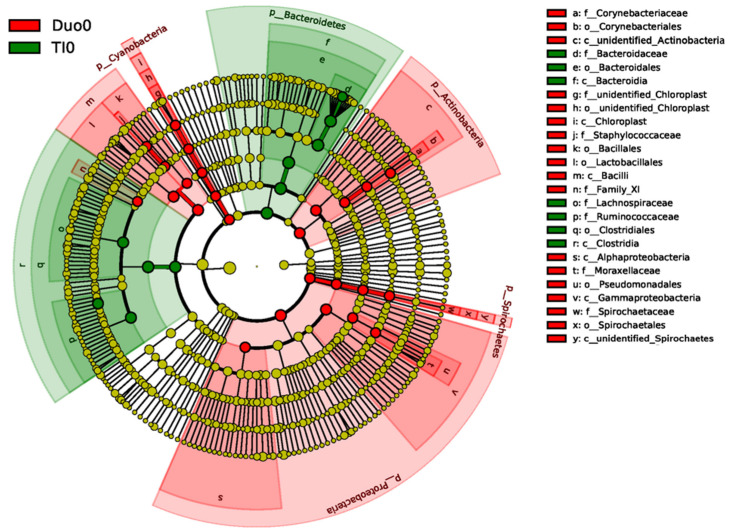
Linear Discriminant Analysis Effect Size (LEfSe) analysis results comparing duodenal and terminal ileum biopsies. The cladogram compares significant differences between species in the duodena of controls (red) and the TI controls (green). Species nodes in yellow are those that are not significantly different. The innermost nodes begin at the phylum level of classification and move outward to class, order, family and genus toward the exterior.

Comparison of specific bacterial OTUs between the control Duo and TI shows a significant increase in abundance of the class of Bacilli, and phyla of Cyanobacteria, Actinobacteria, Spirochetes, and Proteobacteria, in the duodenum. Conversely, the phylum Bacteriodetes, and the class of Clostridia, are significantly more abundant in the TI, compared with the duodena in controls (Figure 4).

### 2.5. Microbial Abundance, Age, Sex, and Villous Length

Age: In our primarily adolescent population, the relative abundance of bacteria did not correlate significantly with age, except Bacteroidetes, which decreased in relative abundance in the TI as age increased (rho = −0.32, *p*-value 0.027). This finding held true in a multivariate analysis that controlled for tissue type (F (1, 43) = 4.14, *p*-value = 0.048).

Sex: Differences in relative abundance were not observed between sexes. The largest difference with respect to sex and relative abundance was found in cyanobateria (F (1, 45) = 4.14, *p*-value = 0.0916), but it did not reach statistical significance.

**Figure 5:**
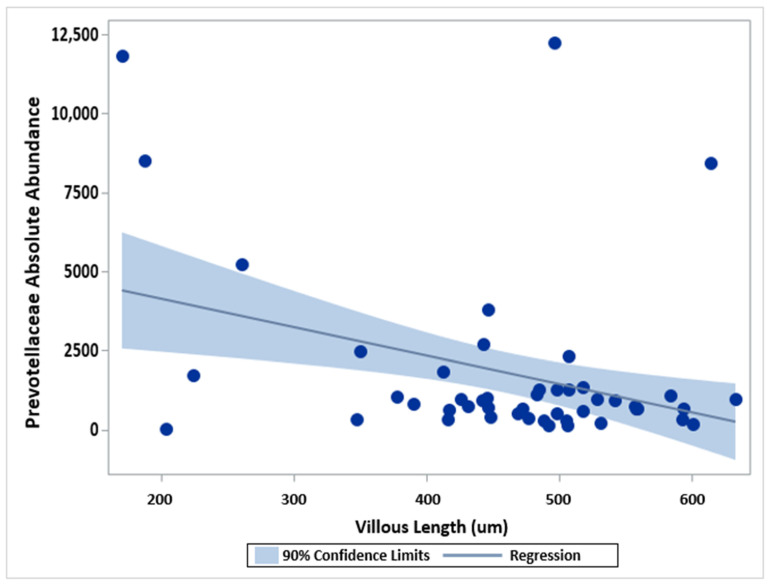
Scatter plot of absolute abundance of Prevotellaceae in all duodenal samples, with duodenal villous lengths fit with a linear regression and 90% confidence interval (shaded area).

Villous Length: As mucosally-adherent bacteria reside on intestinal villi and intestinal villous length is known to vary between inflamed and non-inflamed tissue [13,14], we explored the relationship between bacterial abundance and villous length through Pearson correlation analysis. For both the duodenum and the TI, the median intestinal villous length was shortest in samples from children with CD and active inflammation (Table 1 and Table 2). In the duodenum, a statistically significant negative correlation was observed for villous length and Prevotellaceae abundance (rho = −0.35; *p* = 0.0147) (Table 5). However, marked variability in Prevotellaceae abundance was noted in patients with the shortest villi (Figure 5). No significant correlations were observed between villous length and OTU abundance in the TI (Table 6).

## 3. Discussion

To our knowledge, this study is the first of its kind to characterize the composition of the mucosally-adherent duodenal microbiome in children with and without Crohn’s disease. Such characterization is necessary and represents a pivotal first step in developing targeted treatment options for manipulating the microbiome and restoring microbial balance in the proximal small intestine. This is particularly relevant to children with Crohn’s disease, who are more likely than adults to have duodenal disease involvement [8,9,10,11]. First, our study demonstrates that the pediatric duodenal microbiome of healthy children and children with Crohn’s disease appears distinct from the microbial composition in the TI (Table 2), except for the TI of children with CD without active TI inflammation (TI2 study group). The latter observation is likely due to small sample size in the TI2 study group (*n* = 6), and it is possible that differences in microbial composition may be observed in larger studies. Second, based on our findings, the healthy pediatric duodenal microbiome is characterized by significantly increased relative abundance of Pseudomonadales, compared with children with active duodenal CD, whose duodenal microbiome appears characterized by significantly increased relative abundance of Bacteriodetes, specifically the family Prevotellaceae (Figure 1). These observed differences do not appear to be related to differences in age, sex, or concomitant medication use across or within the duodenal study groups. Although we observed a statistically significantly inverse relationship between villous length and Prevotellaceae abundance in the duodenum (*p* = 0.0147), upon closer inspection of Figure 4, this may be an artifact stemming from the relative sparsity of patients with short villi (<300 µm) and the marked variability in Prevotellaceae abundance within this small subset of patients (*n* = 5). No significant correlations between villous length and abundance of bacterial entities were noted in the TI (Table 6). Larger studies may be needed to tease out the relative contribution of villous length versus active inflammation per se to variability in mucosally-adherent bacterial abundance. We believe such studies are possible, as we have previously demonstrated substantial inter-individual variability in intestinal villous length in children with and without active IBD, such that children with active disease may have intestinal villous length comparable to children with Crohn’s disease in remission or children without Crohn’s disease [14].

While some pediatric studies of microbial composition in the duodenum have been published for other chronic, inflammatory, gastrointestinal conditions such as celiac disease [15], few data exist for IBD. One such study of duodenal aspirates in children with CD vs. controls observed *Atopobium parvulum* relative abundance to be increased in the duodena of children with CD [16], a finding not observed in our study and likely explained by differences in the biospecimen matrix sequenced for microbial rRNA (i.e., duodenal aspirate vs. mucosal biopsy tissue sequenced in our study). Another study of duodenal aspirates demonstrated decreased Firmicutes, Actinobacteria, and *Bacteroidetes* abundance in the duodena of children with UC [17]. The observation of decreased *Bacteroidetes* in the duodena of patients with UC in this prior work by Sjoberg et al., is the opposite of our findings in Crohn’s disease, and consistent with previous observations of known microbiome differences between Crohn’s disease and UC [18]. Differences in study findings may also be explained by the use duodenal aspirates in the UC study, as opposed to our use of mucosal biopsy tissue. Although the QC pass rate for duodenal biopsy samples was lower than for TI biopsy samples, through this study we provide important proof of concept that duodenal mucosal biopsies can be used to characterize microbial composition in the proximal bowel of children. We believe the observed difference in the QC pass rate between the duodenum and TI was likely related to the generally lower abundance of bacteria in the proximal bowel, compared with the distal bowel. With fewer bacteria present in the duodenum than in the TI, the likelihood of bacterial genetic material contamination by host DNA/RNA is greater; hence, the lower QC pass rate.

Similar to findings in other studies, our study demonstrates regional differences in microbial composition across the length of the human intestine. More specifically, our observations of increased Bacteriodetes in the control TI compared to control duodenum are in line with prior work in adults. In a study by Vuik et al., patients undergoing double balloon enteroscopy for undiagnosed abdominal symptoms were biopsied along the entirety of their small intestine, with biopsies submitted for16S rRNA sequencing demonstrating an increased relative abundance of Firmicutes and Bacteriodetes in the TI, and increased Proteobacteria and Firmicutes in the duodenum [7]. This prior work in adults supports our observations in the duodena of children. Although our analysis did not specifically demonstrate a significant difference in the relative abundance of Firmicutes, it did show a significant increase in Bacilli, a bacterial class within the phylum Firmicutes (Figure 4). Our observations of increased clostridia and lachnospiraceae in the TI of controls, compared with active CD, is also in line with prior work in children by Haberman et al. [19]. The consistency of our observations in the TI with prior adult and pediatric studies of the mucosally-adherent microbiome [7,19] lends credibility to our methodologies and our novel microbiome findings in the pediatric duodenum.

Interestingly, our observations of decreased Bacteriodetes in healthy vs. inflamed duodena are the opposite of previous observations in the TI made by Haberman et al. [19]. Our findings also illustrate increased relative abundance of Pseudomonadales (Proteobacteria phylum) in the duodena of controls vs. active CD, contrary to prior observations in the TI, where Proteobacteria abundance was increased in active IBD [19]. Notably, only TI samples without active inflammation from patients with evidence of active colonic CD were included in the study by Haberman et al. [19], which may help explain the difference between our study findings and theirs. Alternatively, regional differences in the normal healthy composition of the microbiome in the duodenum vs. TI may explain this trend reversal between the two disease locations in the small intestine in active CD. The significance of this reversal in abundance between the duodenum and TI remains uncertain and warrants further investigation to help determine what roles Proteobacteria may play in inflammatory pathogenesis versus disease protection. Our findings suggest that the role of these OTUs in microbial homeostasis may differ depending on the location within the small intestine.

Comparing our study findings to other works describing the ileal mucosal microbiome, Assa et al. demonstrated an increase in OTU abundance of *Faecalibacterium prausnitzii* (genus of Ruminococcus and class Clostridia) in a small study of 10 pediatric patients with newly diagnosed CD compared with controls [20]. We observed the opposite trend in our larger pediatric cohort, where controls (*n* = 33) demonstrated increased Clostridia abundance compared with children with active CD in the TI (*n* = 29). The abundance of *F. prausnitzii* and Ruminococcus, specifically, was not observed to be significantly different between controls and children with active CD jn the TI in our study. Sequencing of different bacterial regions between studies (V4 in our study vs. V6 in the study by Assa et al.) could contribute to this difference in study findings.

Previous studies have demonstrated differences in microbial composition of the mucosally-adherent microbiome in the TI of active vs. inactive CD, as well as controls [19]. In our study, we noted a significant difference in the overall bacterial composition in the TI of Controls vs. children with active CD, but we did not find significant differences in the TI microbial composition between active and inactive CD (Table 2). These observations may be due to the limitations of the small sample size for the CD Inactive study group (*n* = 6), as differences were detected in the two larger study groups in the TI (Control TI *n* = 33, CD Active TI *n* = 29), but not for the smaller CD Inactive study group. Similarly, the lack of differences in the overall bacterial composition across the three duodenal study arms is likely due to the small sample sizes in CD Active (*n* = 8) and CD Inactive (*n* = 13). Another potential limitation in our study is the lack of available information regarding the typical diet history for individuals enrolled in this investigation, as diet is a known contributor to microbial differences in the human intestine [21]. However, we believe this is mitigated by an important strength of our investigation design. By specifically analyzing the mucosally-adherent microbiome, we aimed to minimize, as much as possible, potential contamination of the sequenced microbiome by confounders such as xenobiota from the diet, which may remain residually in luminal secretions and could be inadvertently collected during duodenal aspiration.

Previous studies of the intestinal microbiome have shown that environmental exposures, such as diet and medication use, alter microbial composition [22,23]. As expected, concomitant medication use was significantly higher in children with CD vs. controls in our investigation, likely due to the need for symptom management of previously undiagnosed or inadequately treated disease in the CD population. However, examination of the contribution of individual drugs (e.g., antibiotics, probiotics, acid suppressors) to variability in the microbiome in the duodenum and TI revealed no significant differences between those receiving and not receiving individual medications (Table 1 and Table 4). Given the relatively small sample size in our study, we were likely underpowered to detect the influence of concomitant medication use on variability in microbial composition in the small intestine. The cross-sectional design of our study limited the ability to determine past antibiotic or probiotic use that may still have affected the microbiome composition on the day of biopsy. We also acknowledge that detailed diet information, which was not collected as part of this investigation, could also contribute to explaining the microbial findings observed. In attempt to minimize the confounding influence of diet, all patients were fasting at the time of biopsy and we did not include any patients following a specialized IBD diet. Another potential limitation of our study is the use of biopsies from children with functional GI symptoms for comparison with IBD, instead of biopsies from healthy, asymptomatic volunteers. However, the nature of pediatric research makes obtaining biopsies for research purposes from asymptomatic healthy children unethical.

Although our study is limited by a relatively small sample size of 73 children, it provides important proof of capability for sequencing microbial rRNA from pediatric mucosal biopsies from the duodenum, where microbial abundance is significantly lower than in the TI or the colon. Our findings suggest that trends in bacterial abundance in health and active Crohn’s disease may be reversed between the duodenum and TI, at least for members of the phylum Proteobacteria. In contrast to observations in the adult and pediatric TI, dysbiosis associated with duodenal Crohn’s disease is characterized by an increased relative abundance of Bacteriodetes, compared with pediatric controls. Studies such as ours represent an important step in understanding the role of specific OTUs in microbiome homeostasis and are essential for developing rational therapeutic strategies for targeted microbiome manipulation for the treatment of Crohn’s disease in the small intestine. This is particularly important for children, who are more likely than adults to be affected by the upper tract disease [8,9,10,11].

## 4. Materials and Methods

### 4.1. Participants

Potential study participants were identified via review of the clinical endoscopy schedule and the electronic medical record (EMR) at the Children’s Mercy Hospital (CMH), a tertiary regional pediatric hospital in the midwestern United States. The subjects were recruited on the day of procedure, prior to endoscopy. All the subjects were fasting at least 8 h for procedural purposes as part of routine medical care. Only those subjects who provided informed consent (if 18 years of age or older), or informed assent with parental/legal guardian consent (if under 18 years of age) were included. All research activities were approved by the CMH Institutional Review Board and conducted in accordance with the ethical standards of the Declaration of Helsinki. In order to be considered for study inclusion, children had to be between 2 and 18 years of age (inclusive), undergoing both upper and lower endoscopy with biopsies for clinical purposes, have a reasonable clinical suspicion for a new diagnosis of IBD or another clinical indication for undergoing endoscopy (e.g., abdominal pain), and not receiving systemic immunomodulating, immunosuppressive, or biological drugs. Children with an established diagnosis of IBD who were not yet receiving treatment with systemic immunomodulating, immunosuppressive, or biological agents were eligible to enroll. All concomitant medication use at the time of endoscopy was recorded.

The diagnosis of Crohn’s disease was established by agreement by two independent, experienced, pediatric gastroenterologists on review of the EMR post-endoscopy, and two independent, experienced, pediatric pathologists on histopathologic review of biopsies collected for clinical purposes. Based on the histopathologic review, the Crohn’s disease patients were sorted into study arms based on evidence of active inflammation in the duodenal biopsies (Duo1) versus active inflammation elsewhere within the GI tract but normal duodenal histology (Duo2). TI study arms were designated using the same approach. Once the cohort of children with Crohn’s disease was finalized, sex-matched and age-matched (within 12 months) controls without IBD were selected from a pool of enrolled children who were ultimately diagnosed with functional/psychosomatic etiologies, without evidence of inflammation or other histopathology (e.g., celiac disease) at any of the sites biopsied for clinical purposes (i.e., esophagus, stomach, small and large intestine). Assessment and agreement on control designation (Duo0 for duodenal and TI0 for TI biopsies) was performed by the same two pathologists and gastroenterologists as above.

### 4.2. Sample Collection

Up to four mucosal biopsies were collected for study purposes from the duodenum and TI from all participants, in close proximity to the location of biopsies obtained for clinical purposes. Fresh tissue samples collected for research purposes were flash-frozen and stored appropriately at −70 °C until batch analysis.

### 4.3. DNA/RNA Extraction and Microbial Sequencing

Using the Allprep DNA/RNA/miRNA extraction kit (Qiagen, Valencia, CA, USA) according to manufacturer’s recommendations, total DNA was extracted from intestinal mucosal biopsy samples collected for study purposes. The DNA quantity was determined with a NanoDrop ND-1000 spectrophotometer (ThermoFisher Scientific, Waltham, MA, USA). Total DNA (150 ng) was submitted to a commercial service provider for analysis of sequence variation in the 16S ribosomal RNA (rRNA) bacterial gene region V4 (Novogene; Beijing, China).

Amplicon Generation: 16S rRNA genes of the V4 region were amplified using primer 515F-806R. All the PCR reactions were carried out with Phusion^®^ High-Fidelity PCR Master Mix (New England Biolabs, Ipswich, MA, USA).

PCR Products quantification and qualification: Equal volumes PCR product and 1X loading buffer (containing SYB green) were mixed for electrophoresis on 2% agarose gel for detection. Samples with the bright main strip between 400 and 450 base pairs (bp) were chosen for further experiments.

PCR Products Mixing and Purification: The PCR products were mixed in equidensity ratios. Then, the mixture of PCR products was purified with Qiagen Gel Extraction Kit (Qiagen, Hilden, Germany).

The initial quality control analysis revealed some contamination from host genomic DNA, which was not unexpected. The tissue samples were subjected to an additional clean-up step to remove contaminating host genomic DNA. All the samples that passed the quality control analysis were subsequently utilized for library preparation.

Library Preparation: Sequencing libraries were generated usingNEBNext Ultra DNA Library Pre^®^ Kit for Illumina following the manufacturer’s recommendations, and index codes were added. The library quality was assessed on the Qubit@ 2.0 Fluorometer (Thermo Scientific, Waltham, MA, USA) and Agilent Bioanalyzer 2100 system. Finally, the library was sequenced on an Illumina platform Hiseq 2500 and 250 bp paired-end reads were generated.

### 4.4. Villous Length Measurement

The mucosal microbiome adheres to intestinal villi, the length of which varies with age, location along the GI tract, and inflammation/degree of blunting from disease [13,14]. In order to explore relationships between bacterial abundance and intestinal villous length, Infinity Analyze Software (Teledyne Lumenera, Ottawa, ON, Canada) was used to measure the villous length, as previously described by our group [14]. Briefly, the distance from the villous tip to its base, where it curves into the crypt, was measured in micrometers by drawing a line between these two points.

### 4.5. Data Analysis

Descriptive statistics were generated for all included study participants and tissue samples. Microbial OTU data generated by Novogene was assessed for relative bacterial abundance, alpha and beta composition, and overall diversity using Unifraq dissimilarity coefficients and Linear Discriminant Analysis Effect Size (LefSe). A *p* value of <0.05 was considered statistically significant for all analyses.

The overall composition of the microbiome was compared across the various study groups, in both the terminal ileum and the duodenum. The contribution of potential exposures to concomitant antibiotics, probiotics, acid suppressors and other non-systemic immunomodulators to differences in microbial composition was also assessed through Fisher’s Exact tests and two-sided *T*-tests. The influence of sex, age, and villous length was also evaluated this way. Additionally, villous length across the disease subgroups was compared in the duodenum and TI using Kruskal–Wallis tests. Since mucosally-adherent bacteria reside on intestinal villi, the relationships between intestinal villous lengths and microbial abundance in each of the study groups in the Duo and TI were explored using Pearson correlation in order to interrogate the potential effect of villous blunting on CD microbiome diversity and abundance. Lastly, multivariate analysis was performed to investigate microbial abundance variations between the controls and CD while controlling for age, sex, concomitant medication use, and villous length. Analyses were completed using Novogene’s analytics, and additionally through SAS Version 9.4 (SAS Institute, Cary, NC, USA).

## 5. Conclusions

This is the first study of its kind to characterize and compare the mucosally-adherent microbiome of the duodenum in pediatric patients with and without Crohn’s disease. Based on our data, the pediatric duodenum demonstrates increased Pseudomonadales and Spirochetes in healthy mucosa compared with those patients with active duodenal CD, who demonstrate increased Bacteroidetes.

## Figures and Tables

**Table 1:** Baseline characteristics of children with and without Crohn’s Disease (CD) in the duodenum. This study population was comprised of three subgroups based on disease activity: Control (no CD; Duo0), CD-active (active disease in the duodenum; Duo1), CD-inactive (diagnosis of CD without active disease in the duodenum; Duo2). SD—standard deviation; IQR—interquartile range; NA—not applicable.

Variables	Duodenal Study Group			*p* Value (Duo0, 1, 2)	*p* Value (Duo0, 1)
	Control [Duo0 (N = 27)]	Active CD [Duo1 (N = 8)]	Inactive CD [Duo2 (N = 13)]		
Sex				0.207	1.000
Female	12 (44.4%)	3 (37.5%)	2 (15.4%)		
Male	15 (55.6%)	5 (62.5%)	11 (84.6%)		
Age (years) (mean (SD))	13.39 (3.69)	13.70 (2.73)	13.52 (2.36)	0.960	0.984
Age (years) (median (IQR))	14.25 (11.75, 16.17)	13.92 (12.29, 15.88)	13.58 (12.92, 15.58)	0.960	0.984
Villous length (μm) (median (IQR))	498 µm (443.00, 557.00)	242 µm (195.00, 435.50)	485 µm (448.00, 518.00)	0.003	0.003
Concomitant medications	9 (33.3%)	6 (75%)	10 (76.9%)	0.013	0.051
Acid suppression	5 (18.5%)	2 (25.0%)	1 (7.7%)	0.577	0.648
5-ASA	0 (0.0%)	0 (0.0%)	1 (7.7%)	0.437	N/A
Antibiotics	0 (0.0%)	1 (12.5%)	2 (15.4%)	0.077	0.229
Probiotics	1 (3.7%)	0 (0.0%)	3 (23.1%)	0.078	1.000

**Table 2:** Comparison of similarity in overall bacterial composition in the duodenum (Duo) and terminal ilium (TI) across all study groups (ANOSIM analysis). Duo0 & TI0-Controls, Duo1 & TI1-Active CD at the indicated location in the intestinal tract, Duo2 & TI2-CD without active inflammation at the indicated location in the intestinal tract. Data presented as a ratio between within-group and between-group dissimilarities; R-values and *p*-values (in parentheses). An R-value close to 1.0 suggests dissimilarity between groups, while an R-value close to 0 suggests an even distribution of high and low ranks of dissimilarities within and between groups. R-values below 0 suggest that dissimilarities are greater within groups than between groups [12].

Study Group	Duo0 N = 27	Duo1 N = 8	Duo2 N = 13	TI0 N = 33	TI1 N = 29	TI2 N = 6
Duo0	1	−0.003 (0.49)	−0.038 (0.735)	0.558 (0.001)	0.301 (0.001)	0.123 (0.102)
Duo1	-	1	−0.099 (0.948)	0.695 (0.001)	0.321 (0.007)	0.070 (0.189)
Duo2	-	-	1	0.668 (0.001)	0.318 (0.001)	0.028 (0.352)
TI0	-	-	-	1	0.068 (0.017)	0.31 (0.033)
TI1	-	-	-	-	1	0.047 (0.342)
TI2	-	-	-	-	-	1

**Table 3:** Descriptive statistics for the TI sample population comprised of three study groups: Control (TI0), CD-active (TI1), and CD-inactive (TI2). SD—standard deviation, IQR—interquartile range.

		No Medication		Medication		*p*-Value
Group	OTU	N	Absolute Abundance Mean (std. dev)	N	Absolute Abundance Mean (std. dev)	
Duo0	Prevotellaceae	18	1469.00 (1942.94)	9	768.00 (791.71)	0.3116
	Bacteroidales		8934.28 (9890.48)		13,089.78 (20794.81)	0.4836
	Bacteroidia		8938.56 (9891.18)		13,095.89 (20802.23)	0.4836
	Pseudomonadales		5330.78 (6685.56)		2682.33 (2566.65)	0.2660
Duo1	Prevotellaceae	2	2628.00 (3669.88)	6	4097.17 (4838.70)	0.7130
	Bacteroidales		5987.50 (8122.54)		21,602.67 (17,613.49)	0.2883
	Bacteroidia		5987.50 (8122.54)		21,609.00 (17,613.25)	0.2883
	Pseudomonadales		451.00 (545.89)		872.00 (1145.87)	0.6466
Duo2	Prevotellaceae	3	833.33 (474.88)	10	2062.20 (3620.96)	0.5808
	Bacteroidales		15,216.67 (13,674.53)		13,459.40 (15,400.62)	0.8629
	Bacteroidia		15,226.00 (13,689.13)		13,473.20 (15,395.57)	0.8629
	Pseudoonadales		426.00 (157.43)		2974.20 (4620.23)	0.3743

**Table 4:** Descriptive statistics for the TI sample population comprised of three study groups: Control (TI0), CD-active (TI1), and CD-inactive (TI2). SD—standard deviation, IQR—interquartile range.

Variables	TI Study Group			*p* Value (TI0, 1, 2)	*p* Value (TI0, 1)
	Control [TI0 (N = 33)]	Active CD [TI1 (N = 29)]	Inactive CD [TI2 (N = 6)]		
Sex				0.496	0.245
Female	15 (45.5%)	9 (31.0%)	2 (33.3%)		
Male	18 (54.5%)	20 (69.0%)	4 (66.7%)		
Age (years) (mean (SD))	13.70 (3.51)	13.00 (2.55)	11.25 (6.17)	0.238	0.163
Age (years) (median (IQR))	14.25 (11.92, 16.25)	13.00 (11.58, 15.09)	11.74 (8.25, 15.17)	0.238	0.163
Villous length (um) (median) Missing	502.00 (458.00, 552.50) 5	241 (97.50, 367.50) 1	468.00 (444.00, 490.00) 1	<0.001	<0.001
Concomitant Medications	14 (42.4%)	20 (69.0%)	5 (83.3%)	0.044	0.036
Acid Suppression	7 (21.2%)	4 (13.8%)	3 (50.0%)	0.113	0.445
5-ASA	0 (0.0%)	3 (10.3%)	0 (0.0%)	0.204	0.097
Budesonide	0 (0.0%)	1 (3.4%)	0 (0.0%)	0.515	0.468
Antibiotics	0 (0.0%) 33 (100.0%)	3 (10.3%) 26 (89.7%)	1 (16.7%) 5 (83.3%)	0.063	0.097
Probiotics	1 (3.0%)	3 (10.3%)	1 (16.7%)	0.199	0.332

**Table 5:** Pearson correlation analysis of duodenal villous length and bacterial OTU abundance. Correlation coefficient reported between each OTU and villous length, with *p* value reported in parentheses.

Pearson Correlation Coefficients, N = 48 *p*-Value					
	Villous length	Prevotellaceae	Bacteroidales	Bacteroidia	Pseudomonadales
Villous Length	1.00000	−0.35 (0.0147)	−0.11 (0.4669)	−0.11 (0.4671)	0.05 (0.7324)
Prevotellaceae		1.00000	0.51 (0.0002)	0.51 (0.0002)	−0.12 (0.4578)
Bacteroidales			1.00000	1.00 (<0.0001)	−0.24 (0.0947)
Bacteroidia				1.00000	−0.24 (0.0947)
Pseudomonadales					1.00000

**Table 6:** Pearson correlation analysis of TI villous length (when available) and bacterial OTU abundance. Correlation coefficient reported between each OTU and villous length, with *p* value reported in parentheses.

Pearson Correlation Coefficients *p*-Value Number of Observations					
	Villous length	Prevotellaceae	Bacteroidales	Bacteroidia	Pseudomonadales
Villous Length	1.00000 61	0.21871 (0.0904) 61	0.10402 (0.4250) 61	0.10398 (0.4252) 61	−0.11788 (0.3656) 61
Prevotellaceae	0.21871 (0.0904) 61	1.00000 68	0.23623 (0.0524) 68	0.23622 (0.0525) 68	0.06367 (0.6060) 68
Bacteroidales	0.10402 (0.4250) 61	0.23623 (0.0524) 68	1.00000 68	1.00000 (<0.0001) 68	−0.33609 (0.0051) 68
Bacteroidia	0.10398 (0.4252) 61	0.23622 (0.0525) 68	1.00000 (<0.0001) 68	1.00000 68	−0.33608 (0.0051) 68
Pseudomonadales	−0.11788 (0.3656) 61	0.06367 (0.6060) 68	−0.33609 (0.0051) 68	−0.33608 (0.0051) 68	1.00000 68

## Data Availability

Data is contained within the article.
